# Development of a social media-based intervention targeting tobacco use and heavy episodic drinking in young adults

**DOI:** 10.1186/s13722-019-0141-9

**Published:** 2019-04-01

**Authors:** Danielle E. Ramo, Meredith C. Meacham, Manpreet Kaur, Ella S. Corpuz, Judith J. Prochaska, Derek D. Satre

**Affiliations:** 1 Department of Psychiatry, Weill Institute for Neurosciences, University of California, 350 Parnassus Avenue, Box 0984, San Francisco, CA 94143 USA; 2 Department of Medicine, Stanford Prevention Research Center, Stanford University, Stanford, CA USA; 3 Division of Research, Kaiser Permanente Northern California, Oakland, CA USA; 4 Hopelab, San Francisco, CA USA

**Keywords:** Facebook, Smoking, Drinking, Alcohol, Young adults, Social media, Focus groups

## Abstract

**Background:**

Tobacco use and heavy episodic drinking (HED) commonly co-occur in young adults. We developed and tested usability of the Smoking Tobacco and Drinking (STAND) intervention for young adults delivered on Facebook.

**Methods:**

To inform the intervention, focus groups were held with 25 young adults age 18 to 25 (12% female; Mean age = 20.4) who smoked cigarettes and reported at least one HED episode in the past month. Facebook intervention posts (N = 180) were tailored to readiness to quit smoking, and tested in two private Facebook behavioral change groups (*Ready, Not Ready)* with N = 29 young adults (10% female; Mean age = 20.8). Participants flagged posts in need of change, and we assessed engagement (comment frequency).

**Results:**

Focus groups revealed preference for changing one substance at a time and greater receptivity to quitting smoking than reducing drinking. Mean comments per post were 5.3 (*SD* = 1.1) in Ready groups and 11.7 (*SD* = 5.1) in Not Ready groups; 94/180 (52.2%) posts were flagged for change. The level of engagement and the flagging of posts for change did not differ by group or by whether the post targeted tobacco, alcohol, or both substances combined (all p > .10). Overall, STAND was rated as easy to understand, providing sound advice, worthy of recommendation, and helpful (all agreement 100% among Ready; 50–70% among Not Ready).

**Conclusions:**

The current findings informed development of a social media-based intervention targeting tobacco and alcohol use in young adults. Although there was greater interest in making changes in smoking than drinking behavior, receptivity and acceptability of the Facebook post messages in the STAND intervention was high overall. The intervention is being further refined for evaluation in a larger trial.

*Trial registration* *Name of the registry* Smoking Tobacco and Drinking Study (STAND); *Trial registration number* NCT03163303; *Date of registration* 5/23/17; *URL of trial registry record* https://clinicaltrials.gov/ct2/show/NCT03163303.

**Electronic supplementary material:**

The online version of this article (10.1186/s13722-019-0141-9) contains supplementary material, which is available to authorized users.

## Background

The co-occurrence of smoking and drinking is high among young adults, and complicates intervention efforts to reduce use of both substances. Up to 98% of college students drink [1], and smoking is especially common among heavy drinkers and binge drinkers [2]. In 2016, 24% of young adults reported smoking tobacco and 38% reported heavy episodic drinking (HED; 4+ drinks for women and 5+ drinks for men in a day) at least once in the past 30 days [3]. The co-use of tobacco and alcohol, as well as co-occurrence of nicotine dependence and alcohol use disorder, are more common among young adults than any other age group and more common among men than women [4]. Tobacco use poses a risk for subsequent heavy drinking among young adults [5], and using both substances makes it more difficult to quit either one [6–9]. Reaching the U.S. Healthy People 2020 national public health goals of cutting the smoking rate to < 12% and binge drinking to < 24% by 2020 [10] will require novel approaches to reach and intervene with young adults who engage in both of these health risk behaviors, especially those without health insurance or who lack access to traditional alcohol or tobacco use treatment programs.

Addressing tobacco use and HED simultaneously can lead to better outcomes in young adults than addressing each substance separately. Previous digital health intervention research has shown that integrated treatment for tobacco and alcohol resulted in better tobacco and alcohol outcomes for young adults than treatment of tobacco alone [11, 12]. Extending an integrated intervention to a digital environment could maximize reach and utility for young adults. Two previous studies have tested the effectiveness of a digital health intervention jointly targeting smoking and HED in young adults [13]. Witkiewitz and colleagues developed a 14-day mobile feedback intervention based on the Brief Alcohol Screening and Intervention for College Students (BASICS) curriculum that resulted in a decrease in the number of cigarettes smoked compared to a minimal assessment control condition but did not reduce HED or concurrent smoking and drinking at the 1-month follow-up. In a study of students in Switzerland, a 3-month mobile intervention demonstrated no overall beneficial effects for an integrated smoking and alcohol cessation intervention compared to smoking cessation alone. However, participants with high-risk alcohol consumption in the integrated intervention decreased their cigarette use to a much greater extent than those in the single intervention [14]. More research is needed to determine how best to harness digital tools to target tobacco use and HED in young adults.

Interventions using social media represent a promising strategy to deliver evidence-based treatment for smoking and HED to young adults. Social media is extremely popular among young adults and can be harnessed to influence a broad range of health behaviors including smoking cessation [15–17]. Previous evaluations using social media to change health risk behaviors have shown feasibility as measured by participants’ engagement and satisfaction [18–26] and short-term efficacy, especially in the area of smoking cessation [27, 28]. Although prior studies have examined how alcohol-related posting on social media is related to consumption, social-media based alcohol interventions have not been tested [29–31]. Existing alcohol-focused interventions have been targeted mainly at college students and have been conducted via websites or smartphone applications rather than through social media [32–34].

Among young adults in the United States who are online, 87% have a Facebook account and 70% of those with accounts use them daily [35]; hence, there is great promise that Facebook could be used to deliver public health interventions. Furthermore, Facebook is an online space that is already visited on a regular basis (as opposed to a separate webpage or mobile application) and has platform-specific features that facilitate creating secret groups, enabling interactions between participants, and integrating contact with a trained counselor. Secret Facebook groups are a particularly unique feature with respect to privacy protections in that membership, content, and even the existence of the group is not accessible for those not invited to the group by the study or group administrators.

Our group previously developed the *Tobacco Status Project (TSP)*, a Facebook smoking cessation intervention for young adults guided by the Transtheoretical Model of behavior change [36] for which participants were recruited using advertisements on Facebook. Tailored to readiness to quit smoking, TSP demonstrated feasibility with 21% of participants reporting 12-month quit rates [37]. A randomized controlled trial [38] (N = 500) comparing the intervention to referral to a smoking cessation website showed significant post-treatment differences in biochemically-verified abstinence from smoking, but these differences were not sustained at 12 months follow-up [39]. While promising in the area of tobacco treatment, addressing HED in a social media intervention could both strengthen the tobacco intervention and address frequently co-occurring health risk behaviors among young adults.

To guide the development of a social media intervention targeting smoking and HED, it is imperative to examine the target population’s views about tobacco, heavy alcohol use, and co-use; interest in a social media-based intervention; receptivity to intervention content; and barriers to engagement (e.g., privacy concerns). In this study, we present formative work (focus groups), intervention development processes, and usability testing of the *Smoking Tobacco and Drinking (STAND)* intervention on Facebook, in a multi-phased approach.

## Methods

Intervention development progressed through a three-stage process: (1) an exploratory phase in which focus groups were conducted on Facebook with the target population; (2) a development phase based on our prior work and outcomes from focus groups; and (3) a usability-testing phase that included pilot testing of the intervention on Facebook. All study procedures were approved by the University of California, San Francisco (UCSF) Institutional Review Board (IRB).

### Focus groups

Online focus groups with young adults who smoke and engage in HED helped to guide intervention design [40]. Focus groups have the advantage of bringing rich, qualitative data to a design process [41, 42], and have been used in digital health intervention research to gain perspective from “lay people” in the target population in addition to the frame of reference of clinical researchers [43].

Our team’s methods for conducting online focus groups with young adult smokers have been described in detail previously [40]. A focus group guide was developed with 43 open-ended questions about tobacco and alcohol use, tobacco and alcohol co-use, social media use, and intervention preferences based on our previous work with young adults who use substances [44]. Young adults ages 18 to 25 who had smoked ≥ 100 cigarettes in their lifetime, currently smoked on 4 or more days of the week on average, and reported at least HED episode in the past month (4+ drinks for women; 5+ drinks for men) were recruited online via Facebook. Based on prior work [44], we aimed to sample 20–30 participants in at least two focus groups. We conducted three groups in total with 5 to 11 participants per group. Eligibility and consent were assessed online and those eligible were sent a survey electronically that assessed: (1) Sociodemographics: age, ethnicity, individual household income, years of education, and employment status; (2) Smoking behavior: average number of cigarettes smoked per day, average number of days smoking per week, nicotine dependence with the Fagerström Test of Cigarette Dependence (FTCD) [45], age of initiation of smoking, presence of at least one purposeful quit attempt of 24 h or more in the past year, and readiness to quit smoking assessing motivation and importance to quit on a scale of 1–10; [46] (3) Drinking behavior: age of initiation of drinking, average number of drinks consumed per drinking day, past-month HED frequency, the Alcohol Use Disorders Identification Test-Consumption (AUDIT-C) [47] and readiness to change alcohol use assessing motivation and importance to quit on a scale of 1–10.

Survey completers were invited to join one of three private Facebook groups. The first post in the group introduced the moderators and asked participants to say hello. Subsequent posts reminded participants of the study purpose and instructions for responding to question posts. These three 90-min focus groups were moderated by the PI (first author) and study coordinator (third author), with the study coordinator posting content from the focus group guide and the PI asking follow-up questions and encouraging elaboration on responses. Several minutes elapsed between posting each new question so as not to overwhelm participants and to allow time for them to respond. Participants were instructed to answer all questions at their own pace and also were invited to comment on the answers of other users if desired. After completion of the groups, all data were extracted from Facebook and the groups were closed. Participants were sent a $20 Amazon gift card for their participation. Two research staff members independently developed a list of codes using a directed content analysis approach, in which major themes on any topic were identified from the interviews and grouped into concepts. A final version of the coding guide was developed after data were compared, any discrepancies were resolved between the two coders (in consultation with the principal investigator if needed). Researchers also examined themes separately for women and men, noted very few differences in the most frequent terms used, and opted to pool codes for overall data interpretation.

This process was facilitated by using Dedoose [48], a cross-platform web application for analyzing qualitative and mixed methods data. Themes were displayed using word clouds; examination of specific quotes illustrated themes and were used to inform intervention development.

### Theoretical models and intervention design

The overall design of the intervention was based on our group’s TSP Facebook smoking cessation intervention [37–39], which has three main features: (1) assignment of participants to secret (i.e. entirely private and not visible to non-members) Facebook behavior change groups tailored to their readiness to quit smoking (*Ready* in the next 30 days or *Not Ready* in the next 30 days), (2) delivery of Facebook posts each day for 90 days, and (3) weekly “The Dr. Is In” live group sessions with a PhD level counselor. During these sessions the counselor provided limited content for discussion and participants could ask questions and receive support using Facebook commenting features based on Motivational Interviewing and cognitive behavioral coping skills training.

For the current intervention content to be usability tested, posts targeting tobacco cessation was based on the U.S. Clinical Practice Guidelines for smoking cessation [49] and the Transtheoretical Model (TTM) of behavior change [46]. Additional posts targeting alcohol were based on the National Institute on Alcohol Abuse and Alcoholism (NIAAA) *Rethinking Drinking* website and brochure [50] and the *Guide to Alcohol Screening and Brief Intervention for Youth* [51]. Alcohol posts assessed drinking behavior, provided normative feedback, and utilized cognitive and behavioral strategies to support reduction in heavy drinking. A third group of posts addressed combined smoking and HED, including awareness of the prevalence and risks of co-use, identifying triggers related to co-use, and suggesting that participants attempt strategies to reduce drinking and cut down on or quit smoking simultaneously.

### Usability testing

Usability testing in the form of user-feedback and evaluation of key indicators of engagement and outcomes (e.g., smoking and drinking) is imperative to ensure that an intervention achieves “specified goals with effectiveness, efficiency, and satisfaction in a specified context of use.” [52] Usability testing has been described as an integral part of the developmental process for many digital health interventions [53–55].

#### Participants and procedures

Participants were recruited through a Facebook advertisement campaign from 04/03/2017 to 04/10/2017 that targeted young adults in the United States. Participants eligible for the usability study were aged 18 to 25, smoked ≥ 100 cigarettes in their lifetime and currently smoked ≥ 4 days/week, engaged in at least one HED episode in the past month, and used Facebook ≥ 3 days/week. In a similar process as that used for the focus groups, participant eligibility determination and informed consent were completed online, including questions designed to assess understanding of study risks. Those eligible were sent a baseline assessment with the same sociodemographic, smoking, and alcohol use measures as those used in the focus group survey. Stage of change for quitting smoking was recoded from the question about readiness to quit smoking in the next 30 days into readiness to quit within the next 6 months or sooner (*Ready/Not Ready*).

Upon completing the baseline assessment, all participants were invited to one of two “secret” (entirely private) Facebook groups based on their readiness to quit smoking (*Ready*/*Not Ready*). Three posts to each group were made daily for a period of 30 days. Participants were asked to respond to the questions asked in the post (e.g., “What are some of your favorite personal qualities? How does smoking fit (or not fit) with that image of you?”), and were also asked to give open-ended feedback about the design/content of the post (see Fig. 2 for a sample). Two researchers moderated the groups. The moderators replied to the comments, answered any questions participants had, and encouraged participants to elaborate on specific feedback. All participants were sent a follow-up survey that included a usability questionnaire [37] along with the same questions asked in the baseline assessment. All data were extracted from Facebook and the two secret groups were closed. The comments were tallied and all participants who commented on at least two thirds of posts were sent a $20 gift card. Participants were also sent $20 for each survey assessment, for a total possible compensation for study participation of $60.

#### Data extraction/analyses

Posts were divided into quartiles based on the week of the month they were presented to participants and the total number of comments was tallied and z-transformed within the context of its quartile. This transformation was applied to account for a general reduction in commenting over time, as observed in the feasibility trial for the Tobacco Status Project [56]. A data extraction guide was developed by two independent coders in consultation with the principal investigator in a manner similar to that used in the focus groups. A single study staff member then used the extraction guide to code each post and all comments on the following criteria: (1) The number of content-relevant responses (i.e., number of comments responding directly to a question asked in the posts); (2) Number of negative comments (i.e., the number of comments related to design or content that were judged to be negative); (3) User suggestions for post revision (any user-suggested revisions to design and/or content); and (4) A number flagging a post for revision based on the coders judgement (a post was flagged 0 = no change, 1 = change, 2 = delete). All posts that were flagged for change or deletion with negative feedback were reviewed by the team for modification.

Additional analyses then examined the number of posts that were flagged for change/deletion and the level of engagement (comment volume) in the Ready and Not Ready groups. Within each group, the number of posts flagged for change/deletion and the level of engagement were examined by substance type targeted by the post (alcohol, tobacco, or both) using Chi square and ANOVA tests. Finally, usability and satisfaction ratings of the intervention were evaluated by tallying the proportion of users answering “agree” or “disagree” to each item on the usability measure.

## Results

### Focus groups

**Fig. 1:**
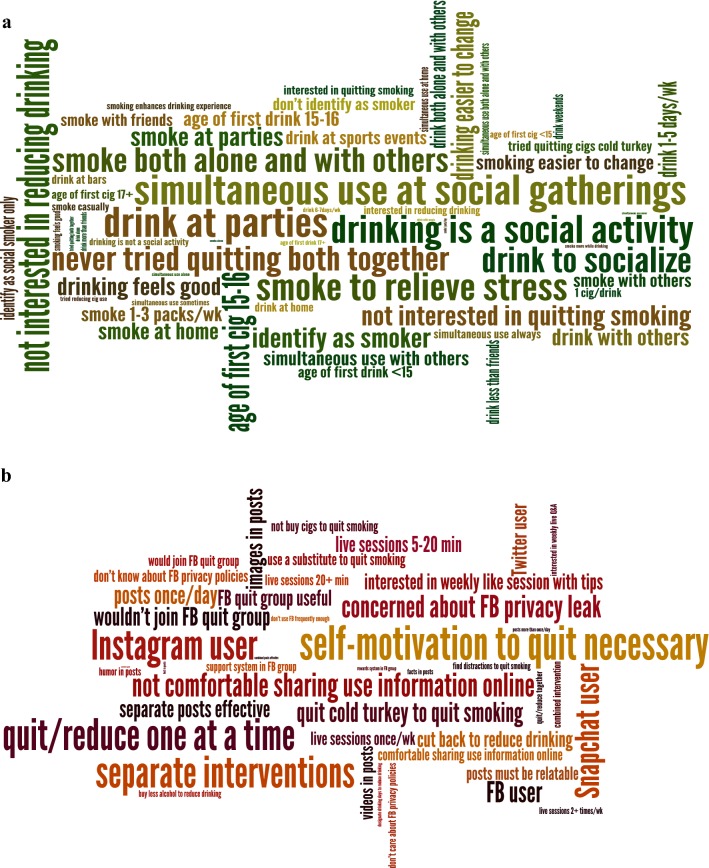
**a** Most common codes pertaining to alcohol and tobacco use patterns, **b** most common codes pertaining to social media use and social media to quit tobacco or change alcohol use. Size of word represents frequency of code use

A total of 25 individuals (age *M* = 20.4, *SD* = 1.9; 12% Female; 72% non-Hispanic White) participated in the three online focus groups (Table 1). Responses to open-ended questions from focus group participants fell into four broad categories: contexts of smoking and drinking; changing smoking and HED; joining a Facebook quit group; and suggestions for a Facebook quit group. Figure 1a represents the most common themes coded pertaining to substance use patterns; Fig. 1b represents the frequency of themes pertaining to social media use in general by young adults and use of social media to quit tobacco or change alcohol use (Additional file 1: Table S1).

**Table 1:** Demographic characteristics of participants in the STAND focus groups and usability testing

	Focus groups (N = 25)		Usability testing (N = 29)	
	N or Mean	% or SD	N or Mean	% or SD
Age (M, SD)	20.4	1.9	20.8	2.0
Current gender identity				
Male	21	84%	24	83%
Female	3	12%	3	10%
Other	1	4%	2	7%
Race/ethnicity (N, %)				
Non-Hispanic White	18	72%	21	72%
Hispanic/Latino	2	8%	3	10%
Asian	1	4%	1	3%
Black	0	0%	3	10%
American Indian/Alaskan Native	1	4%	0	0%
Other	3	12%	1	3%
Household income (N, %)				
$20,000 or less	6	24%	5	17%
$20,001–60,000	8	32%	11	38%
$60,001–100,000	2	8%	8	28%
Above $100,000	10	36%	5	17%
Years of education (M, SD)	13.3	1.3	12.8	2.7
Currently enrolled in school (N, %)				
Full-time school	7	28%	11	38%
Part-time school	3	12%	0	0%
Employment status (N, %)				
Employed full time (> 20 h/wk)	4	16%	14	48%
Unemployed (looking for work)	13	52%	5	17%

#### Contexts of combined smoking and drinking

Drinking and smoking was reported most commonly in social contexts including with friends and at parties. One participant considered the influence of others: “*Nothing like a nice drunk cig. About half the times I drink, I also end up smoking a few cigarettes. This only happens when I am with other people.*” [Focus Group (FG)-3, M19 (male age 19)]. This person also recalled his first smoking experience: “*…I was at a frat party with some new friends, and one of them offered me a cigarette. We were drunk, I gave it a try. It made me cough a ton but it made my body feel good and I was relaxed*” [Focus Group (FG)-3, M19 (male age 19)].

#### Changing smoking and HED

While participants shared examples of trying to quit smoking, attempts were generally short and relapse often occurred in a drinking situation. For example: “*I’ve ‘quit’ a few times. Basically, it’s just me not smoking for a month or whatever but after whatever amount of time I’ll end up back smoking at parties or whatever* [57].” [Focus Group (FG)-3, M19 (male age 19)]. No participants had ever tried using an online group to quit. Participants were more receptive to quitting smoking than changing any aspect of their drinking (e.g., reducing the amount they drink overall, refraining from HED), and preferred an intervention targeting one substance over a combined intervention as demonstrated by this participant’s declaration: “*I don’t plan on changing my drinking habits until the end of college. At that point, I will drink however much my job allows me to*” [Focus Group (FG)-3, M20 (male age 20)].

#### Joining a Facebook behavior change group

Participants had mixed reactions to joining a group on Facebook targeting smoking and/or drinking. Their main concerns were related to hesitation about sharing challenges with others, as mentioned by this participant: “*Not everyone wants to share their struggles and stuff*.” [Focus Group (FG)-2, M22 (male age 22)] Another concern was perceiving low likelihood of success, such as a participant who revealed: “*I’ll be happy to give my opinion, but at the end of the day, I know it won’t change me*.” [Focus Group (FG)-2, M23 (male age 23)]. An additional concern related to sharing information online. While most participants reported that they were unaware of or unconcerned about Facebook privacy policies, there were some concerns about sharing information about substance use, such as this participant who explained: “*…I generally try to keep incriminating things away from the internet. I don’t see FB as a private space whatsoever*” [Focus Group (FG)-3, M19 (male age 19)].

#### Suggestions for Facebook posts and behavior change groups

There were many suggestions as to what would be appealing in an intervention. Receiving one social media post daily that would address smoking and drinking separately in different posts was considered ideal. One participant reasoned: “[I’d like to see posts] *daily, to keep myself on track; but not more than 1*-*2 times/day, as to not overload the user*” [Focus Group (FG)-1, M20 (male age 20)]. Another participant explained: “*…Yea, there’s a correlation between smoking and drinking, but I don’t know much about the causal relationship. I would prefer to target each problem separately*” [Focus Group (FG)-1, F24 (female age 24)]. Most participants were inclined toward seeing pictures and videos in posts, stating “*videos and pictures would probably be more convincing than words*” [Focus Group (FG)-3, M19 (male age 19)]. Almost all participants were interested in live sessions with a counselor and suggested availability of once a week for 20–30 min. One participant stated: “*…this would be the most helpful aspect of having a FB group. A Q&A is a good way to deal with the situation in an interactive way…*” [Focus Group (FG)-3, M19 (male age 19)].

### STAND intervention

Based on the emerging themes from the focus groups and our theoretical framework, the STAND intervention was developed. The intervention included 90 posts each for *Ready* and *Not Ready* groups. Posts included content that targeted tobacco only (55% of *Ready* posts; 31% of *Not Ready* posts); alcohol only (24% of *Ready* posts; 45% of *Not Ready* posts); and both substances (20% of *Ready* posts; 24% of *Not Ready* posts). Tobacco-only content was more common in the *Ready* groups given the preparation stage of group members for changing their tobacco use and to tie into active behavior change strategies. Alcohol-only content was more common in the *Not Ready* groups and targeted at resolving ambivalence and enhancing motivation to change both alcohol and tobacco behaviors given our focus group findings that most participants were not ready to change their alcohol behavior.

The TSP intervention was already tailored to readiness to quit smoking, though this content was sometimes modified to have more updated images or fonts. Messages for *Ready* posts focused on developing a plan for quitting and preventing relapse, while messages for *Not Ready* posts focused more on assessing and enhancing motivation for quitting. Based on the results of focus groups showing few participants were ready to change multiple substances at the same time, and most desired to see content separately for alcohol and tobacco, alcohol content was centered on motivational interviewing principles. For example, alcohol content highlighted potential health- and social relationship-related harms from drinking as well as potential benefits of cutting back (e.g., “Have you ever gotten into a physical or verbal altercation while drinking that could have been avoided? How might you have handled it differently?”, “What would be the best thing that could happen if you drank less?”). In contrast, participants generally indicated greater readiness to reduce tobacco smoking and so while tobacco content in *Not Ready* groups was also tied to motivational interviewing principles, tobacco content in *Ready* groups was focused on helping participants identify and implement specific strategies to help them stop smoking.

Images for posts came from stock photo websites and were related to the content of the message in the post (e.g., images of young people engaging in healthy active behaviors) or contained popular internet content (e.g., cute or funny animals). Images of cigarettes and alcohol were avoided when possible to try to avoiding triggering participants to smoke or drink.

Despite the preponderance of men recruited for focus groups, we aimed to develop post wording that was equally appealing to the experiences of men and women, and we observed no gender differences in reported acceptability of the posts or revision suggestion patterns. Previous technology-aided intervention research has have found preferences that differ by gender [58] and our work suggested that the intervention should be equally appealing and relevant for both genders.

### Usability testing

Initially, 66 people were eligible to participate in the usability testing and out of those 37 completed the baseline survey. All 37 participants were invited to participate in the usability groups. Finally, a total of 29 individuals (*Mean age* = 20.8, *SD* = 2.0, 10% Female; Table 1) participated in usability-testing (21 *Not Ready* to quit smoking; 8 *Ready* to Quit smoking; Table 2). In the *Not Ready* group, 38% (8 out of 21) commented on two thirds of posts and 76% (16 out of 21) completed the follow-up survey. In the *Ready* group, 63% (5 out of 8) commented on two thirds of posts and 88% (7 out of 8) received payment for completing the follow-up survey.

**Table 2:** Smoking and drinking characteristics of participants in the STAND focus groups and usability testing

	Focus groups (N = 25)		Usability testing (N = 29)	
	N or Mean	% or SD	N or Mean	% or SD
Smoking characteristics				
Age of initiation (M, SD)	15.3	2.6	15.1	3.4
Daily smoker (N, %)	24	96%	20	69%
Number of smoking days per week (M, SD)	4.8	2.2	6.2	1.7
Number of cigarettes per smoking day (M, SD)	5.6	6.9	11.1	11.5
FTCD: smoke within first 30 min of waking (% yes)	6	24%	6	21%
Any past year quit attempt	19	76%	18	62%
Smoking stage of change (N, %)				
Precontemplation	10	40%	10	34.5%
Contemplation	11	44%	11	37.9%
Preparation	4	16%	8	27.6%
Drinking characteristics				
Age of initiation (M, SD)	14.4	2.8	15.1	2.4
AUDIT C: alcohol quantity (N, %)				
1 or 2	10	40%	7	24.1%
3 or 4	6	24%	10	34.5%
5 or 6	4	16%	6	20.7%
7 or 9	3	12%	3	10.3%
10 or more	2	8%	3	10.3%
AUDIT C: alcohol frequency (N, %)				
Never	0	0%	1	3.4%
Monthly or less	4	16%	1	3.4%
2–4 times a month	2	8%	2	6.9%
2–3 times a month	3	12%	5	17.2%
2–3 times a week	7	28%	10	34.5%
4 or more times a week	9	36%	10	34.5%
AUDIT C total score (M, SD)	10.2	2.9	10.1	2.3
Past month HED episodes (≥ 4 for women, ≥ 5 for men) alcoholic drinks (M, SD)	8.8	6.1	10.1	7.7
Alcohol stage of change (N, %)				
Precontemplation	13	52%	10	34.5%
Contemplation	3	12%	7	24.1%
Preparation	2	8%	2	6.9%
Action	3	12%	7	24.1%
Maintenance	4	16%	3	10.3%
Quit importance (M, SD)	3.8	2.7	5.7	3.0
Confidence to quit (M, SD)	7.4	2.7	6.2	3.2
Long term goals about alcohol use (N, %)				
Abstain	0	0%	2	6.9%
Drink lightly and moderately	16	64%	21	72.4%
Thoughts about drinking less (N, %)				
Never think of drinking less	11	44%	7	24.1%
Sometimes think of drinking less	8	32%	12	41.4%
Decided to drink less	1	4%	1	3.4%
Already trying to cut back	5	20%	9	31%

*HED* heavy episodic drinking, *FTCD* Fagerstrom test for cigarette dependence, *AUDIT* alcohol use disorders identification test

#### Usability and satisfaction with the STAND intervention

**Fig. 2:**
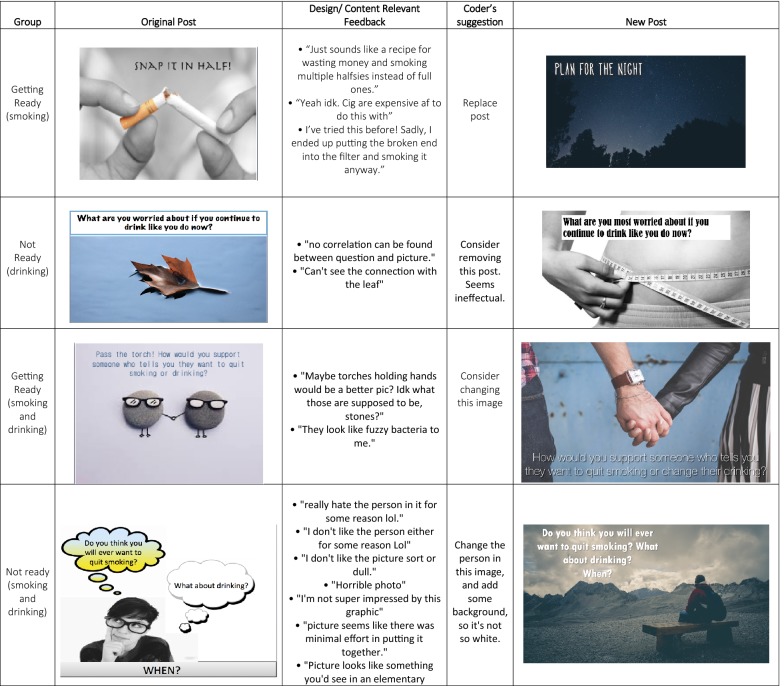
Sample of post feedback adaptation based on usability feedback

**Fig. 3:**
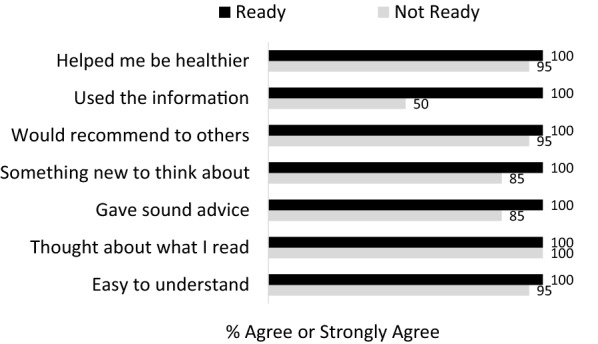
Intervention usability ratings by participant level of readiness to quit

Participants rated the extent to which study treatment materials were engaging and useful (Fig. 3) and made suggestions about how to clarify and adapt posts (Fig. 2). Overall, the STAND intervention was rated by 91% (22/24) participants as easy to understand, by 83% (20/24) as providing sound advice, by 87% (21/24) as a program they would recommend to others, and 62% rated the posts overall as helpful (15/24), with ratings slightly higher among those ready to quit (Fig. 3). Additionally, 70% (17/24) reported that they clicked on the informational links embedded in the posts. However, 54% (94/180) of posts were flagged for some type of change.

## Discussion

We found that social media (i.e., Facebook) was a useful platform to conduct formative work needed to adapt an evidence-based online tobacco cessation treatment for young adults to address both tobacco use and HED. Focus groups conducted with young adults who smoked cigarettes and reported recent HED helped to inform strategies for addressing both tobacco use and HED in the context of a single intervention. Although content for targeting smoking (e.g., U.S. clinical practice guidelines [49], our own Facebook intervention work [37]) and heavy drinking (e.g., NIAAA rethinking drinking project) [50] among young adults was available, it was unclear how best to address both substances in a social media context.

While many specifics of the current intervention remained the same (e.g., frequency of daily posting, weekly sessions with a counselor), this formative work supported the development of refined intervention content intended to be more engaging to this target population. Focus groups indicated that young adults had different thoughts about their use of the two substances and varied in readiness to change. Focus groups were also useful to determine who might be most easily recruited for a tobacco and HED intervention on Facebook. A key question was whether the content in our existing tobacco intervention should be altered for an intervention addressing both tobacco and HED. Given the focus group recommendations to target each substance individually and to keep post frequency to about one per day, the findings suggested that more tobacco-related content would not be needed and that additional alcohol-related content should be targeted at enhancing motivation rather than changing behavior. We used this information to tailor “combined” posts by identifying how drinking situations may be triggers to smoke, and by addressing situations in which both substances are used socially, such as bars or parties.

Satisfaction and usability testing of the developed intervention through Facebook also provided valuable data to inform further refinement. For example, given that “helpful” ratings were relatively low (62%) in comparison to other ratings, posts were revised to increase the practicality of content related to reducing drinking and smoking. Additionally, low scoring posts were replaced and images were updated to be more closely connected to the prompt and to be more aesthetically pleasing.

Advantages of Facebook included its status as the mostly widely used social media platform (and the medium we planned to use for the final intervention trial), thus supporting feasible evaluation in the context of Facebook behavior change groups. Further, the format of asking participants to respond to study posts and give feedback on posts was well-received, and yielded findings that were helpful in informing which changes should be made before launching the trial.

Since there were no differences in feedback between posts that addressed tobacco, alcohol, or both substances, we did not make major changes in the proportion or strategy of combining information of posts. However, approximately half of posts were deemed in need of revision by the team in response to participant comments. We changed multiple aspects of the posts, including everything from the font and image to the message of individual posts based on specific feedback (i.e., comments on an individual post) and also incorporated more general feedback on the intervention throughout the usability-testing phase (Additional file 2: Figure S1).

Overall, focus groups and usability-testing were each important and useful in the intervention development process. Although focus groups indicated that some people were hesitant to participate in an intervention combining both smoking and HED, usability-testing showed that combined posts were generally well-received by users. Notably, the alcohol-related posts were motivationally focused and did not request explicit action. Further, findings on receptivity to the intervention demonstrated an overall positive reaction to the content and mode of delivery. The development of STAND was innovative in both its efforts to address two high-risk behaviors (smoking and HED) and also its mode of delivery (social media). During the intervention design phase, there were questions about how best to incorporate alcohol content into the existing smoking cessation intervention. Focus groups informed the best way to combine an intervention for these two substances and deliver valuable content. The next phase of this work is an online randomized controlled trial (in process) to determine the efficacy of the STAND intervention compared to the TSP intervention that addresses only tobacco [59].

### Limitations

Sample sizes for the focus groups and usability-testing sessions were fairly small. The low proportion of women in focus groups may mean findings are not generalizable to women who smoke cigarettes and have reported HED. However, qualitative examination of responses by men versus women revealed no differences in overall acceptability of the intervention or recommended revisions to posts. Further, although there were few, if any concerns about Facebook’s privacy policies, it is worth noting that focus groups were conducted before privacy breaches related to the Facebook-Cambridge Analytica data scandal [60]. Incentivizing participants for engagement in usability testing may limit implications about whether participants are likely to engage in the full trial, but nonetheless provided important information about the relative appeal and usefulness of posts compared to one another. Finally, while Facebook is the most widely used social media platform [61], young adults are also heavy users of other tools such as Snapchat and Instagram that are worth exploring as tools for intervention delivery as well.

### Conclusions

Social media has the potential to reach a wide audience of people who may not be willing or able to participate in existing interventions for health behavior change. Yet few models exist to inform treatment development. Our approach used the medium of Facebook for intervention delivery in a multi-phased, mixed-methods approach to data collection and analysis. The next phase of this work will compare treatment outcomes of interventions targeting tobacco alone (TSP) versus tobacco plus HED (STAND) among young adults.

## Additional file

**Additional file 1: Table S1.** Most common content codes from focus groups.

**Additional file 2: Figure S1.** Process for development of the Smoking Tobacco and Drinking (STAND) Study Intervention.

## References

[1] Weitzman ER, Chen YY (2005). The co-occurrence of smoking and drinking among young adults in college: national survey results from the United States. Drug Alcohol Depend.

[2] Gubner NR, Delucchi KL, Ramo DE (2016). Associations between binge drinking frequency and tobacco use among young adults. Addict Behav.

[3] Substance Abuse and Mental Health Services Administration. Key substance use and mental health indicators in the United States: Results from the 2016 National Survey on Drug Use and Health. Rockville, MD: Center for Behavioral Health Statistics and Quality, Substance Abuse and Mental Health Services Administration: NSDUH Series H-52; 2017.

[4] Falk DE, Yi HY, Hiller-Sturmhofel S (2006). An epidemiologic analysis of co-occurring alcohol and tobacco use and disorders: findings from the National Epidemiologic Survey on Alcohol and Related Conditions. Alcohol Res Health.

[5] Harrison EL, McKee SA (2011). Non-daily smoking predicts hazardous drinking and alcohol use disorders in young adults in a longitudinal U.S. sample. Drug Alcohol Depend.

[6] Murray RP, Istvan JA, Voelker HT, Rigdon MA, Wallace MD (1995). Level of involvement with alcohol and success at smoking cessation in the lung health study. J Stud Alcohol.

[7] Hymowitz N, Cummings KM, Hyland A, Lynn WR, Pechacek TF, Hartwell TD (1997). Predictors of smoking cessation in a cohort of adult smokers followed for five years. Tob Control.

[8] Duffy SA, Ronis DL, Valenstein M, Lambert MT, Fowler KE, Gregory L (2006). A tailored smoking, alcohol, and depression intervention for head and neck cancer patients. Cancer Epidemiol Biomark Prev.

[9] Jaszyna-Gasior M, Schroeder JR, Moolchan ET (2007). Alcohol use and tobacco abstinence among adolescents in cessation treatment: preliminary findings. Addict Behav.

[10] U.S. Department of Health and Human Services Office of Disease Prevention and Health Promotion. Healthy People 2020 Washington, DC; 2019 [cited 2019 January 22]. https://www.healthypeople.gov/.

[11] Ames SC, Werch CE, Ames GE, Lange LJ, Schroeder DR, Hanson AC (2010). Integrated smoking cessation and binge drinking intervention for young adults: a pilot investigation. Ann Behav Med.

[12] Ames SC, Pokorny SB, Schroeder DR, Tan W, Werch CE (2014). Integrated smoking cessation and binge drinking intervention for young adults: a pilot efficacy trial. Addict Behav.

[13] Witkiewitz K, Desai SA, Bowen S, Leigh BC, Kirouac M, Larimer ME (2014). Development and evaluation of a mobile intervention for heavy drinking and smoking among college students. Psychol Addict Behav.

[14] Haug S, Castro RP, Kowatsch T, Filler A, Schaub MP (2017). Efficacy of a technology-based, integrated smoking cessation and alcohol intervention for smoking cessation in adolescents: results of a cluster-randomised controlled trial. J Subst Abuse Treat.

[15] Cao B, Gupta S, Wang J, Hightow-Weidman LB, Muessig KE, Tang W (2017). Social media interventions to promote HIV testing, linkage, adherence, and retention: systematic review and meta-analysis. J Med Internet Res.

[16] Pagoto S, Waring ME, May CN, Ding EY, Kunz WH, Hayes R (2016). Adapting behavioral interventions for social media delivery. J Med Internet Res.

[17] Elaheebocus S, Weal M, Morrison L, Yardley L (2018). Peer-based social media features in behavior change interventions: systematic review. J Med Internet Res.

[18] Bull SS, Levine DK, Black SR, Schmiege SJ, Santelli J (2012). Social media-delivered sexual health intervention: a cluster randomized controlled trial. Am J Prev Med.

[19] Patrick K, Marshall SJ, Davila EP, Kolodziejczyk JK, Fowler JH, Calfas KJ (2014). Design and implementation of a randomized controlled social and mobile weight loss trial for young adults (project SMART). Contemp Clin Trials.

[20] Young SD (2013). Social media technologies for HIV prevention study retention among minority men who have sex with men (MSM). AIDS Behav.

[21] Kernot J, Olds T, Lewis LK, Maher C (2013). Effectiveness of a facebook-delivered physical activity intervention for post-partum women: a randomized controlled trial protocol. BMC Public Health.

[22] Cavallo DN, Tate DF, Ries AV, Brown JD, DeVellis RF, Ammerman AS (2012). A social media-based physical activity intervention: a randomized controlled trial. Am J Prev Med.

[23] Napolitano MA, Hayes S, Bennett GG, Ives AK, Foster GD (2013). Using facebook and text messaging to deliver a weight loss program to college students. Obesity.

[24] Pedrana A, Hellard M, Gold J, Ata N, Chang S, Howard S (2013). Queer as F**k: reaching and engaging gay men in sexual health promotion through social networking sites. J Med Internet Res.

[25] Williams G, Hamm MP, Shulhan J, Vandermeer B, Hartling L (2014). Social media interventions for diet and exercise behaviours: a systematic review and meta-analysis of randomised controlled trials. BMJ Open.

[26] Jones K, Baldwin KA, Lewis PR (2012). The potential influence of a social media intervention on risky sexual behavior and Chlamydia incidence. J Community Health Nurs.

[27] Baskerville NB, Azagba S, Norman C, McKeown K, Brown KS (2015). Effect of a digital social media campaign on young adult smoking cessation. Nicotine Tob Res.

[28] Naslund JA, Kim SJ, Aschbrenner KA, McCulloch LJ, Brunette MF, Dallery J (2017). Systematic review of social media interventions for smoking cessation. Addict Behav.

[29] Moreno MA, Whitehill JM (2014). Influence of social media on alcohol use in adolescents and young adults. Alcohol Res Curr Rev.

[30] Hendriks H, Van den Putte B, Gebhardt WA, Moreno MA (2018). Social drinking on social media: content analysis of the social aspects of alcohol-related posts on facebook and instagram. J Med Internet Res.

[31] Boyle SC, LaBrie JW, Froidevaux NM, Witkovic YD (2016). Different digital paths to the keg? How exposure to peers’ alcohol-related social media content influences drinking among male and female first-year college students. Addict Behav.

[32] Bertholet N, Daeppen JB, McNeely J, Kushnir V, Cunningham JA (2017). Smartphone application for unhealthy alcohol use: a pilot study. Subst Abuse.

[33] Bertholet N, Godinho A, Cunningham JA (2018). Smartphone application for unhealthy alcohol use: pilot randomized controlled trial in the general population. Drug Alcohol Depend.

[34] Bhochhibhoya A, Hayes L, Branscum P, Taylor L (2015). The use of the internet for prevention of binge drinking among the college population: a systematic review of evidence. Alcohol Alcohol.

[35] Greenwood S, Perrin A, Duggan M. Social Media Update 2016: Pew Research Center: Internet, Science, and Technology; 2016 [cited 2016 November 29]. http://www.pewinternet.org/2016/11/11/social-media-update-2016/ Archived by Webcitation at http://www.webcitation.org/6vglS0nRR.

[36] Prochaska JO, DiClemente CC (1983). Stages and processes of self-change of smoking: toward an integrative model of change. J Consult Clin Psychol.

[37] Ramo DE, Thrul J, Chavez K, Delucchi KL, Prochaska JJ (2015). Feasibility and quit rates of the tobacco status project: a facebook smoking cessation intervention for young adults. J Med Internet Res.

[38] Ramo DE, Thrul J, Delucchi KL, Ling PM, Hall SM, Prochaska JJ (2015). The Tobacco Status Project (TSP): study protocol for a randomized controlled trial of a Facebook smoking cessation intervention for young adults. BMC Public Health.

[39] Ramo D, Thrul J, Delucchi KL, Hall SM, Ling PM, Belohlavek A, Prochaska JJ (2018). A randomized controlled evaluation of the tobacco status project, a facebook intervention for young adults. Addiction.

[40] Thrul J, Belohlavek A, Hambrick DA, Kaur M, Ramo DE (2017). Conducting online focus groups on Facebook to inform health behavior change interventions: two case studies and lessons learned. Internet Interv.

[41] Klassen AC, Creswell J, Plano Clark VL, Smith KC, Meissner HI (2012). Best practices in mixed methods for quality of life research. Qual Life Res.

[42] Zhang W, Creswell J (2013). The use of “mixing” procedure of mixed methods in health services research. Med Care.

[43] Yardley L, Morrison L, Bradbury K, Muller I (2015). The person-based approach to intervention development: application to digital health-related behavior change interventions. J Med Internet Res.

[44] Ramo DE, Liu H, Prochaska JJ (2014). A mixed-methods study of young adults’ receptivity to using facebook for smoking cessation: if you build it, will they come?. Am J Health Promot.

[45] Heatherton TF, Kozlowski LT, Frecker RC, Fagerström KO (1991). The fagerström test for nicotine dependence: a revision of the Fagerström Tolerance Questionnaire. Br J Addict.

[46] Prochaska JO, DiClemente CC (1983). Stages and processes of self-change for smoking: toward an integrative model of change. J Consult Clin Psychol.

[47] Bush K, Kivlahan DR, McDonell MB, Fihn SD, Bradley KA (1998). The AUDIT alcohol consumption questions (AUDIT-C): an effective brief screening test for problem drinking. . Ambulatory Care Quality Improvement Project (ACQUIP). Alcohol Use Disorders Identification Test. Arch Intern Med.

[48] Dedoose Version 4.5, web application for managing, analyzing, and presenting qualitative and mixed method research data. Los Angeles, CA: SocioCultural Research Consultants, LLC (www.dedoose.com); 2013.

[49] Fiore MC, Jaén CR, Baker TB, et al. Treating tobacco use and dependence: 2008 update. Clinical Practice Guideline. Rockville, MD: U.S. Department of Health and Human Services. Public Health Service; 2008.

[50] National Institute on Alcohol Abuse and Alcoholism (2010). Rethinking drinking: alcohol use and your health.

[51] National Institute on Alcohol Abuse and Alcoholism. Alcohol screening and brief intervention for youth: a practitioner’s guide. US Department of Health and Human Services, editor. Rockville, MD: National Institute on Alcohol Abuse and Alcoholism; 2011.

[52] Standard I. Ergonomic requirements for office work with visual display terminals (vdts)–part 11: guidance on usability. ISO Standard 9241-11: 1998. International Organization for Standardization; 1998.

[53] Radovic A, Gmelin T, Hua J, Long C, Stein BD, Miller E (2018). Supporting Our Valued Adolescents (SOVA), a social media website for adolescents with depression and/or anxiety: technological feasibility, usability, and acceptability study. JMIR Mental Health.

[54] Valdés BA, Hilderman CG, Hung C-T, Shirzad N, Van der Loos HM, editors. Usability testing of gaming and social media applications for stroke and cerebral palsy upper limb rehabilitation. In: Engineering in Medicine and Biology Society (EMBC), 2014 36th annual international conference of the IEEE. IEEE; 2014. 10.1109/EMBC.2014.6944402 25570770

[55] Park BK, Nahm E-S, Rogers VE, Choi M, Friedmann E, Wilson M (2017). A facebook-based obesity prevention program for Korean American adolescents: usability evaluation. J Pediatr Health Care.

[56] Thrul J, Klein AB, Ramo DE (2015). Smoking cessation intervention on facebook: which content generates the best engagement?. J Med Internet Res.

[57] Sambunjak D, Straus SE, Marusic A (2010). A systematic review of qualitative research on the meaning and characteristics of mentoring in academic medicine. J Gen Intern Med.

[58] Kim DJ, Choo EK, Ranney ML (2014). Impact of gender on patient preferences for technology-based behavioral interventions. West J Emerg Med.

[59] Ramo DE, Kaur M, Corpuz ES, Satre DD, Delucchi K, Brown SA (2018). Using Facebook to address smoking and heavy drinking in young adults: protocol for a randomized, controlled trial. Contemp Clin Trials.

[60] Granville K. Facebook and Cambridge Analytica: what you need to know as fallout widens. New York Times; 2018.

[61] Pew Internet & American Life Project. Internet User Demographics; 2018 [cited 2015 May 8]. http://www.pewinternet.org/2018/03/01/social-media-use-in-2018/.

