# Supplementary material for: Development of a social media-based intervention targeting tobacco use and heavy episodic drinking in young adults
Source: Addict Sci Clin Pract. 2019 Apr 1;14:14. doi: 10.1186/s13722-019-0141-9 (PMC6444508; doi:10.1186/s13722-019-0141-9)
Supplement: Supplementary file 1 — Additional file 1: Table S1. Most common content codes from focus groups. [file 13722_2019_141_MOESM1_ESM.docx]

**Additional file 1: Table S1. Most common content codes from focus groups**

|  | Alcohol and tobacco use patterns | Use of social media for quitting |
| --- | --- | --- |
| (1) | drink at parties  drinking is a social activity  drink to socialize  simultaneous use at social gatherings  smoke to relieve stress | Instagram user  self-motivation to quit necessary  separate interventions  quit/reduce one at a time |
| (2) | drink to socialize  never tried quitting both together  not interested in reducing drinking  smoke both alone and with others | concerned about FB privacy leak  FB user  not comfortable sharing use information online  Snapchat user |
| (3) | not interested in quitting smoking | quit cold turkey to quit smoking  posts once/day |

*FB: Facebook*
