# Supplementary material for: Development of a social media-based intervention targeting tobacco use and heavy episodic drinking in young adults
Source: Addict Sci Clin Pract. 2019 Apr 1;14:14. doi: 10.1186/s13722-019-0141-9 (PMC6444508; doi:10.1186/s13722-019-0141-9)
Supplement: Supplementary file 2 — Additional file 2: Figure S1. Process for development of the Smoking Tobacco and Drinking (STAND) Study Intervention. [file 13722_2019_141_MOESM2_ESM.docx]

**Final Smoking Tobacco and Drinking (STAND) Study intervention**

**Usability testing** of 90 Facebook posts over 30 days with N=27 participants

**Design of tobacco + alcohol intervention** informed by focus groups + NIAAA guidelines

**Focus Groups** conducted via 3 secret Facebook groups with N=25 participants

**Tobacco Status Project (TSP)** intervention for smoking cessation

**Additional file 2: Figure S1. Process for development of the Smoking Tobacco and Drinking (STAND) Study Intervention.**
